# Peripheral giant cell granuloma in a child with ectrodactyly-ectodermal dysplasia-cleft lip/palate syndrome: a case report

**DOI:** 10.1186/s12903-024-04585-z

**Published:** 2024-08-13

**Authors:** Aman Kumar, Vinay Kumar Srivastava, Sannu Sonal, Vaishali Bhati

**Affiliations:** Faculty of Dental Sciences, Institute of Medical Sciences, Banaras Hindu University, Varanasi, India

**Keywords:** Cleft lip, Ectodermal dysplasia, Peripheral giant cell Granuloma

## Abstract

**Background:**

Ectrodactyly-ectodermal dysplasia-cleft lip/palate (EEC) syndrome mainly affects ectodermal and mesodermal tissues. It is usually manifested as split hands and feet, ectodermal dysplasia, and orofacial clefting, along with other signs and symptoms. A multidisciplinary approach to treatment is required, in which dentists play an important role in identifying and treating various oral conditions that may be genetically linked to or may be the result of EEC syndrome.

**Case presentation:**

The present case describes the oral condition of a young child suffering from EEC syndrome and presenting with peripheral giant cell granuloma (PGCG) in the mandibular anterior region. After obtaining a thorough medical and family history and a clinical examination, the lesion was surgically excised under local anesthesia. The patient was followed up at periodic intervals for the next twenty four months, during which no recurrence of the lesion was observed.

**Conclusion:**

This report highlights the role of a dentist in the management of the oral conditions of patients suffering from EEC syndrome.

## Background

Ectrodactyly-ectodermal dysplasia-cleft lip/palate (EEC) is a rare autosomal dominant genetic disorder due to mutation in TP63 gene [1]. It is characterized by ectrodactyly of distal limbs, ectodermal dysplasia, and cleft lip and palate (cardinal features) [2]. Lacrimal duct abnormalities, renal anomalies, mental retardation, deafness and genitourinary anomalies are various other abnormalities associated with this syndrome [2]. To clinically diagnose a case as EEC syndrome, either two of the three cardinal features should be presented in isolated cases or one of the three cardinal features should be present in familial cases with a first-degree relative affected by the syndrome [3].

Rudiger was the first person to present the case of ectrodactyly, ectodermal dysplasia, and cleft lip and palate malformation, all occurring simultaneously in one patient, and coined the acronym EEC for it; however, he was not able to explain its etiology or its clinical significance [4]. Till now, a very limited number of such cases have been reported in the literature. As it has a reported prevalence of 1 in 90,000 births [5], very scanty information is available about the oral conditions of such patients.

The present case report describes the case of a young child affected by EEC syndrome, its oral manifestations, and the role of a dentist in the overall medical management of such patients.

## Case presentation

**Fig. 1:**
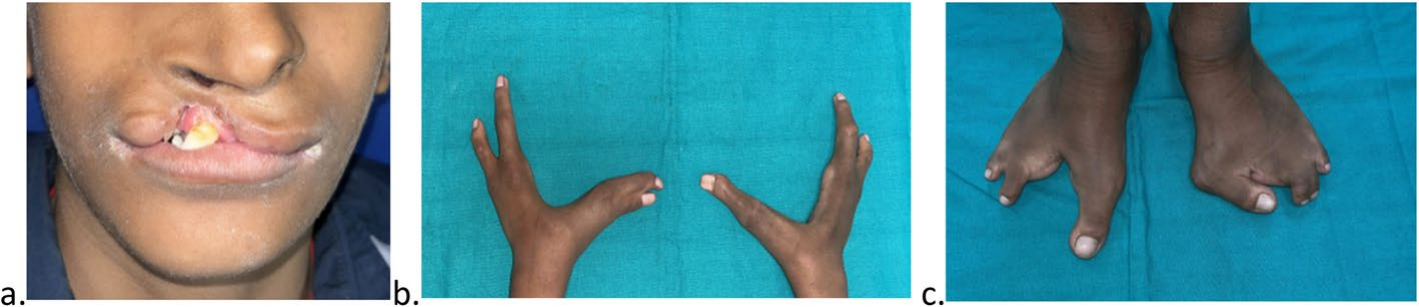
(a) Cleft lip of right side; (b) split hands; (c) split feet

A thirteen-year-old boy presented to the unit of pediatric dentistry with the chief complaint of soft tissue overgrowth in the anterior region of the lower jaw for the last four months. The patient’s mother reported a full-term normal vaginal delivery; no known allergies to the patient were reported, and he was not on any medication. On extraoral examination, a cleft lip was present on the right side, and splitting of both hands and feet was present (Fig. 1). There was a family history of split hand and foot malformations, as the patient’s father was also affected by cleft hands and cleft feet on both sides. The patient had an older sister who was not suffering from this disorder, nor was any other family member affected by this disorder. The patient had a normal density of hair on his scalp, and his nails were also found to be of normal size and shape. There was no previous history of any extraction of permanent teeth.

**Fig. 2:**
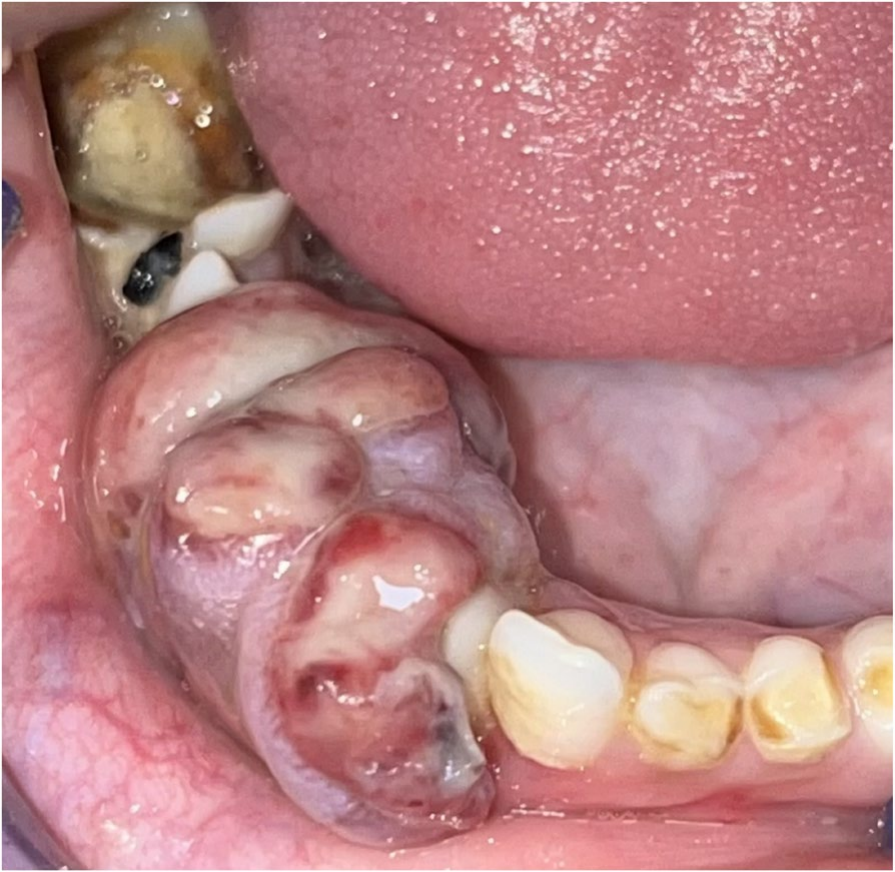
Soft tissue overgrowth over the alveolar ridge in the mandibular anterior region

On intraoral clinical examination, a well-defined pedunculated soft tissue overgrowth was present in the mandibular anterior region, extending from the distal surface of the mandibular lateral incisor of the right side to the second premolar region, cupping over the alveolar ridge (Fig. 2). The overgrowth was soft in consistency and non-tender on palpation. Multiple carious teeth were present. An orthopantomogram (OPG) along with an X-ray of both hands and feet were obtained.

**Fig. 3:**
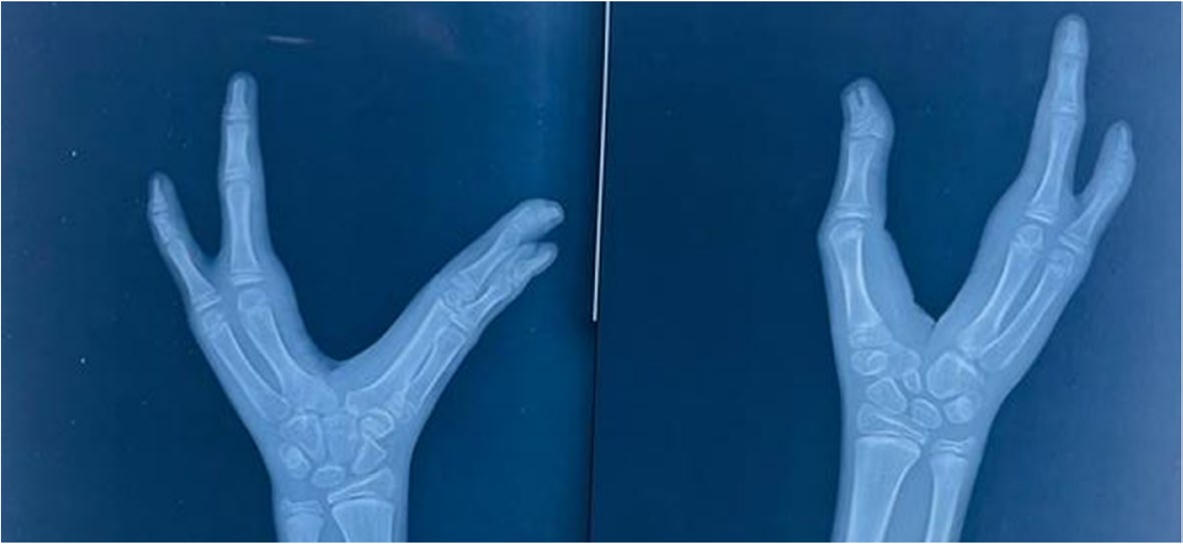
Absence of the third metacarpal along with its phalanges of the left hand and absence of the second and third metacarpals along with their phalanges of the right hand

The X-ray of the left hand revealed an absent third metacarpal and its phalanges. There was fusion of the metacarpals and phalanges of the first and second digits in the left hand. In the right hand, the second and third metacarpals and their phalanges were absent (Fig. 3).

**Fig. 4:**
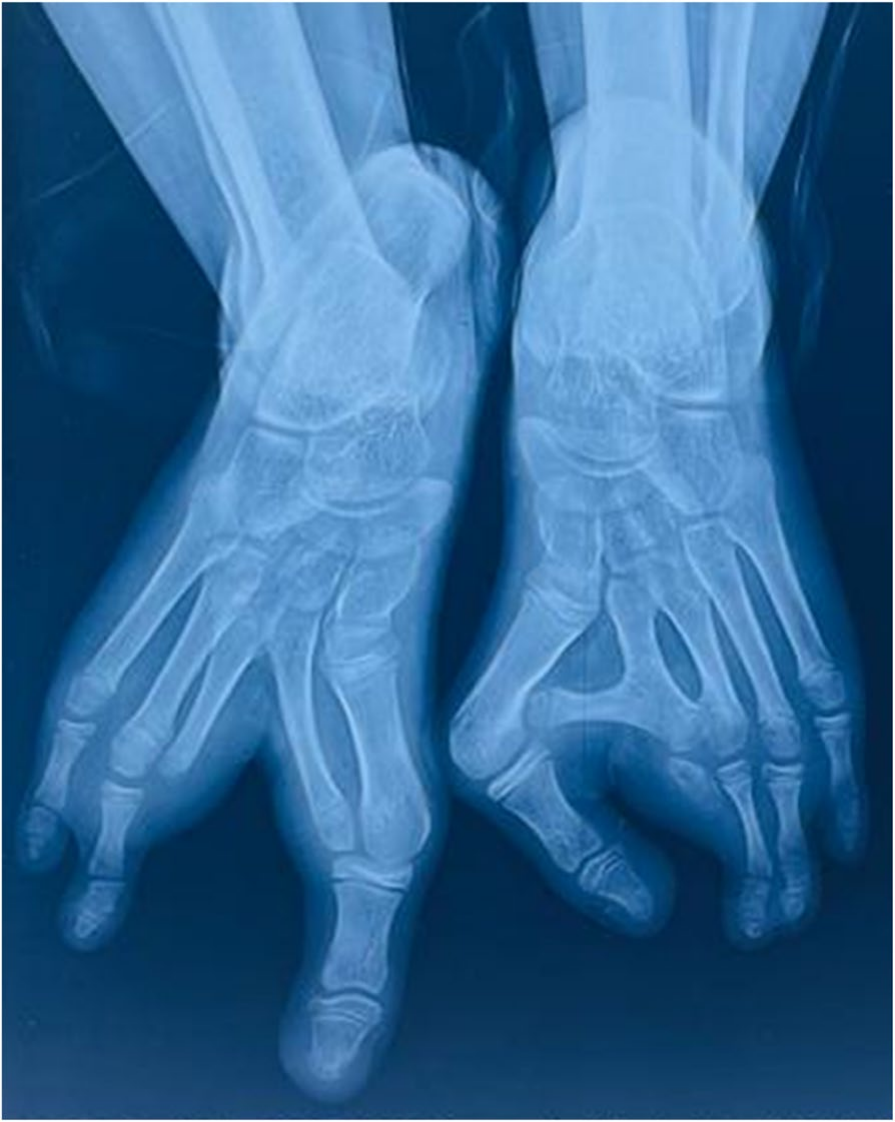
Absence of phalanges of the second and third digits of the right foot and presence of a cross bone between the first and second metatarsals of the left foot

The right foot’s x-ray revealed a cleft between the toe and the fourth digit. The metatarsals of the second and third digits were fused to their adjacent counterparts. The proximal, middle, and distal phalanges of the second and fourth digits were absent. An X-ray of the left foot revealed a cross bone fused to the second metatarsal, causing a deviation of the first metatarsal. The proximal phalanx of the second digit was poorly formed, and there was a complete absence of middle and distal phalanges (Fig. 4).

**Fig. 5:**
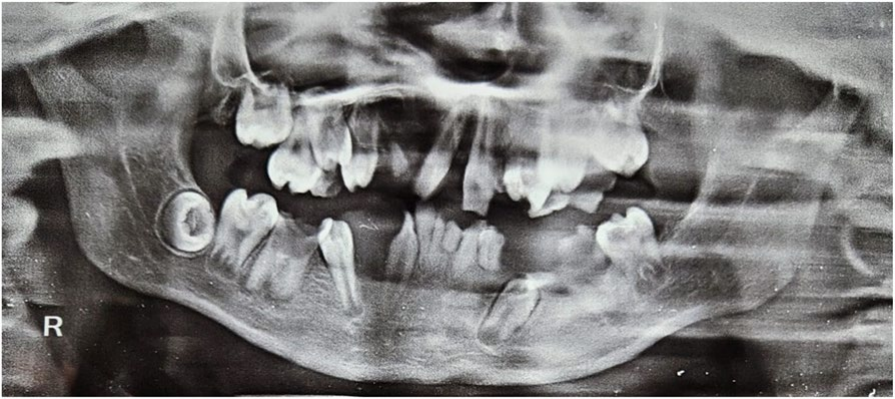
OPG revealing no bony abnormality in mandibular anterior region on right side along with multiple missing permanent teeth

OPG revealed no bony abnormalities in relation to the region of soft tissue overgrowth. Multiple permanent teeth (maxillary lateral incisors, canines, mandibular incisors and premolars) in both the mandibular and maxillary arch were found to be missing (Fig. 5), presenting the oral manifestation of ectodermal dysplasia. Pyogenic granuloma, peripheral giant cell granuloma, peripheral ossifying fibroma, and peripheral cemento-ossifying fibroma were considered in the differential diagnosis.

The patient was referred to the department of pediatrics for systemic evaluation, and no further systemic abnormalities were reported. The reconstructive surgery of the hands and feet to improve their form and function was planned to be performed later.

**Fig. 6:**
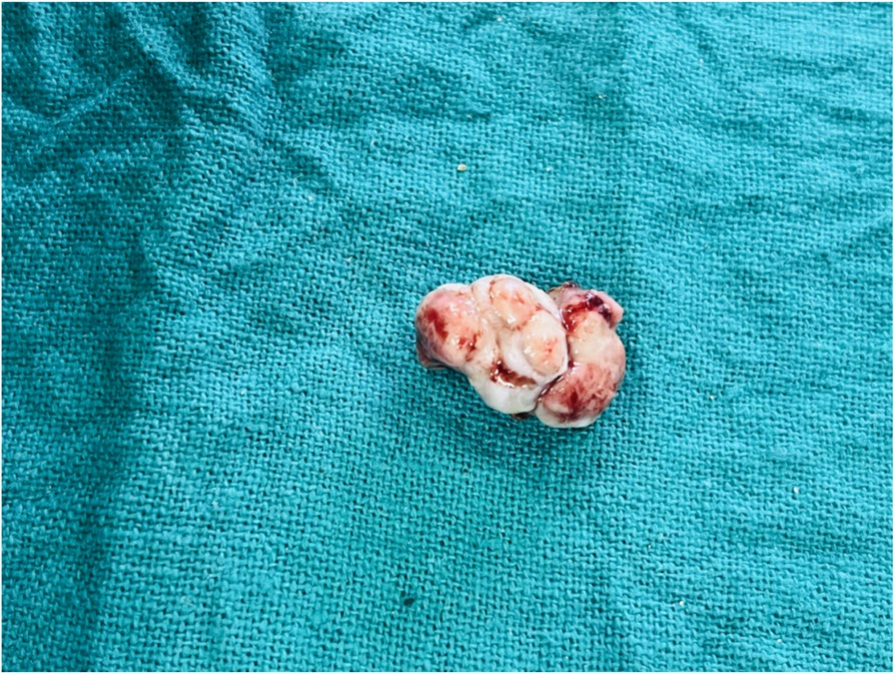
Excised soft tissue overgrowth

**Fig. 7:**
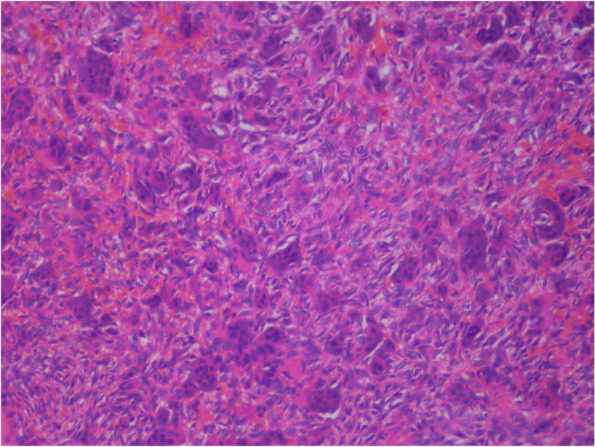
Photomicrograph of excised soft tissue (20X magnification) displaying foreign body giant cells, spindle shaped fibroblasts, extravasated red blood corpuscles

**Fig. 8:**
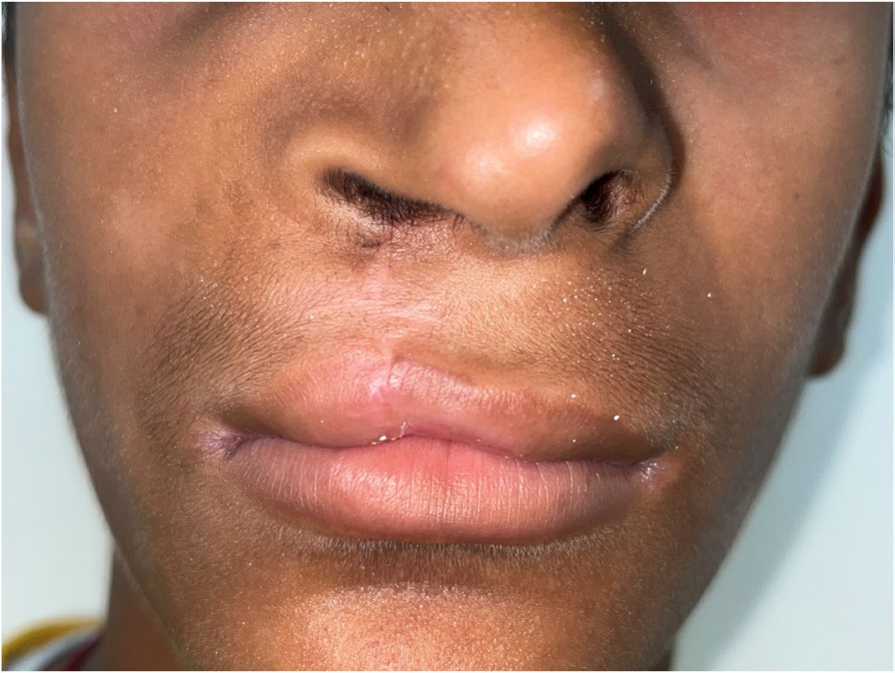
Repaired cleft lip

**Fig. 9:**
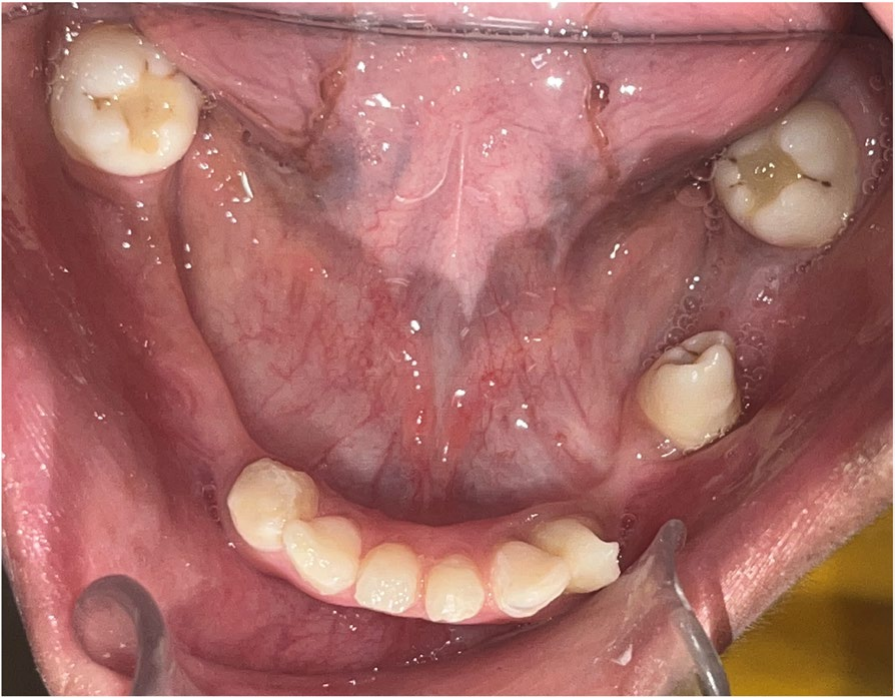
Twenty-four-month follow-up reveals no recurrence of the lesion and complete resolution of the symptoms

**Fig. 10:**
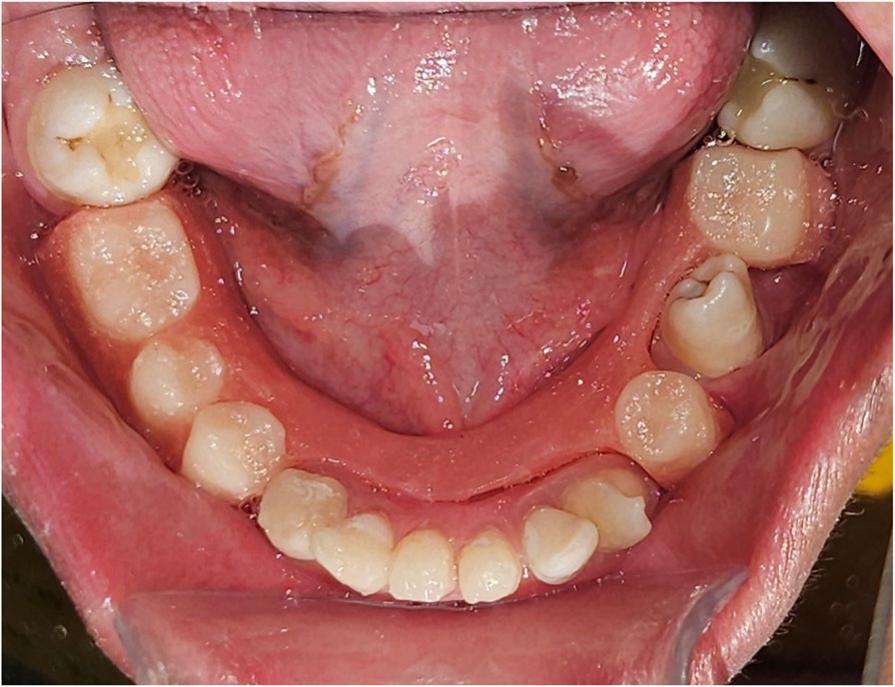
Full mouth rehabilitation and prosthodontic replacement of missing teeth using removable partial denture

The treatment plan of complete excision of the intraoral soft tissue overgrowth was made, which was explained both to the patient and his parents, and informed consent was obtained from the patient as well as his parents prior to the surgical procedure. The lesion was excised under local anesthesia (2% lignocaine with 1:200000 adrenaline) administered by the inferior alveolar nerve block technique, and sutures were placed (Fig. 6). The excised tissue was sent for histopathological analysis, which reported it to be peripheral giant cell granuloma. The histopathological analysis revealed cellular and vascular connective tissue stroma consisting of diffuse foreign body giant cells of varying shapes and sizes containing around twenty nuclei. Stroma contained plump spindle-shaped fibroblasts and numerous endothelium-lined blood capillaries, extravasated red blood corpuscles, and chronic inflammatory cells infiltrate mainly lymphocytes and plasma cells (Fig. 7). The complete oral rehabilitation of the patient was performed by restoring or extracting the carious teeth. Surgical repair of the cleft lip was performed under general anesthesia, which improved the speech and esthetic of the patient (Fig. 8). The patient was further followed up at regular intervals for the next twenty four months, and no recurrence was observed with a complete resolution of symptoms (Figs. 9 and 10).

## Discussion

EEC syndrome is a rare developmental disorder characterized by split hands and feet, ectodermal dysplasia, and a cleft lip and/or palate, leading to significant functional and esthetic problems for the affected individual [2]. The syndrome may present as a sporadic or familial case with variable phenotypic characteristics, which may be due to genetic heterogeneity [6]. The present case reports a familial case of the syndrome with all the cardinal features present.

Split hand and foot malformation (SHFM) can present itself sporadically or as a part of EEC syndrome and is divided into seven subtypes depending upon the molecular and clinical characteristic features [7]. Mutation of the TP63 gene is responsible for 93% of SHFM cases associated with EEC syndrome; however, the exact pathogenesis causing the disrupted phenotype of normal hands and feet is still unclear [7]. Reconstructive surgery of split hands and feet is carried out with the closure of the cleft to improve the functional and cosmetic outcome. In the present case, split hands and feet were present bilaterally, for which reconstructive surgery was planned.

Abnormal development of ectoderm-derived structures is found in ectodermal dysplasia [1]. Commonly affected structures are hair, nails, teeth, sweat glands, etc. In the present case, multiple permanent teeth of the maxillary and mandibular arch were absent, which is a feature of ectodermal dysplasia. The patient also reported intolerance to heat and the absence of sweating, thereby depicting the lack of sweat glands found in hypohidrotic ectodermal dysplasia. Hypomineralized enamel due to ectodermal dysplasia and inefficient tooth brushing due to splint hands may be the reason for multiple carious teeth in the patients suffering from EEC syndrome, as was found in the present case. The chief complaint of the patient in the present case was soft tissue overgrowth in the lower jaw, which turned out to be peripheral giant cell granuloma (PGCG) after histopathological examination. As basal and suprabasal cells of the PGCG epithelium show consistent p63 immunohistochemical reactions [8], mutation of the Tp63 gene may be responsible for the occurrence of PGCG in the patient suffering from EEC syndrome, as it was in the present case. PGCG is not a true neoplasm but rather a reactive lesion with a low recurrence rate after surgical excision if the lesion is completely excised and local irritant factors are completely removed [9]. In the present case, the lesion was completely excised, and no recurrence was observed after twenty four months of periodic follow-up.

Cleft lip and/or cleft palate is one of the common developmental malformations seen in the head and neck region, which may occur either along with other abnormalities as a part of the syndrome or in isolation. Environmental as well as genetic factors, including various genetic mutations, have been identified as etiologic factors for cleft lip and/or palate [10]. In the present case, a cleft lip was present on the right side, for which reconstructive surgery was planned and performed after oral rehabilitation. The follow-up after lip repair showed excellent healing and significant improvement in functional and esthetic outcomes. Prenatal diagnosis of cleft lip and/or palate during routine ultrasonography during pregnancy should be followed by genetic counseling and other investigations to identify other associated features of EEC syndrome [11].

The management of EEC syndrome requires a multidisciplinary approach. Reconstructive surgery of split hands and feet and repair of the cleft lip and/or palate should be carried out at the appropriate age. Dentists play an active role in the management of primary and permanent dentition, the application of topical fluoride, the appropriate management of carious lesions, and other oral conditions that may be linked to this syndrome [12]. Prosthodontic rehabilitation for missing teeth and repair of the cleft lip and palate performed by the dentist in such cases improve the overall oral and systemic health of the patient.

## Conclusion

EEC syndrome presents with a variety of clinical features that present a significant functional and aesthetic challenge to the patients. Early diagnosis is important to provide genetic counseling and appropriate care to the patient. As the condition makes an individual more susceptible to dental caries, regular visits to the dentist are important for the appropriate management of oral conditions. Further research is required to find the genetic linkage between EEC syndrome and various oral conditions.

## Data Availability

All the data is present with the corresponding author (A.K.) which may be made available upon reasonable request.
